# Applicability of imediate, late or serial intact parathyroid hormone measurement following total thyroidectomy

**DOI:** 10.5935/1808-8694.20120012

**Published:** 2015-11-20

**Authors:** Agnaldo José Graciano, Carlos Takahiro Chone, Carlos Augusto Fischer

**Affiliations:** a MD, MSc. in Sciences (Head of the Otorhinolaryngology and Head and Neck Surgery Department at the São José Hospital in Joinville - SC, Brazil); b PhD (Professor of Otorhinolaryngology, Head and Neck at Campinas State University - UNICAMP); c MD (Head and Neck Surgeon and Craniomaxillofacial Surgeon at the São José Hospital in Joinville - SC, Brazil). Hospital São José /Joinville e Disciplina de Otorrinolaringologia Cabeça e Pescoço - UNICAMP

**Keywords:** parathyroid hormone, postoperative complications, thyroidectomy

## Abstract

Hypocalcemia is the most common complication after total thyroidectomy. Intact parathyroid hormone (i-PTH) testing is a proven effective method to detect patients at risk for postoperative symptomatic hypocalcemia. However, there is still uncertainty as to the timing of i-PTH testing in a clinical setting.

**Objective:**

This study looked into the correlation between serum i-PTH levels measured at different times after total thyroidectomy and the risk of symptomatic hypocalcemia.

**Methods:**

This retrospective case series studied a group of 110 consecutive for hypocalcemia and intact parathyroid hormone (PTHi) levels four and twelve hours following total thyroidectomy. Statistical analysis was used to evaluate the performance of isolated and serial i-PTH measurements to determine the likelihood of symptomatic hypocalcemia.

**Results:**

I-PTH is highly sensitive (90.3%-96.8%) and specific (77.2%-87.3%) for symptomatic hypocalcemia. There was no significant difference in the sensitivity levels of the tests done four and twelve hours after surgery or in a serial fashion. However, the 12-hour i-PTH level was more specific (*p* < 0.0007).

**Conclusion:**

Single i-PTH testing done 12 hours after total thyroidectomy may be used as a screening test to detect patients at risk for symptomatic hypocalcemia.

## INTRODUCTION

Hypocalcemia is the most frequent complication in total thyroidectomy and affects 1.7% to 68% of the patients submitted to this procedure. The drop in calcium serum levels is usually transient and often asymptomatic, and correlates with permanent hypoparathyroidism in only 0% to 9% of the cases. However, the presence of symptomatic hypocalcemia may evolve to tetany, seizures, and even death unless it is diagnosed early on and treatment is offered to the patients^1^, ^2^, ^3^, ^4^, ^5^, ^6^, ^7^, ^8^, ^9^. Some authors believe the better way to prevent the risk of hypocalcemia is offering calcium oral supplementation accompanied or not by vitamin D to all patients submitted to total thyroidectomy. However, this approach could mean that 64% to 87% of the patients would be given unnecessary supplementation as they do not develop symptoms and their parathyroid hormone (PTH) levels return to normal within the week after surgery in 70% to 94% of the cases^10^^,^^11^. Additionally, hypercalcemia occurred in 4% of the patients given calcium supplementation and vitamin D after total thyroidectomy^12^, ^13^, ^14^.

Considering the drawbacks of compulsory supplementation, most services resort to clinical monitoring and serial serum calcium level testing to detect the patients with low calcium levels and symptomatic hypocalcemia who could benefit from calcium and vitamin D supplementation. Once most patients present symptoms of hypocalcemia and/or significant drops in serum calcium starting at 48 hours after surgery, with symptoms peaking in as many as four days^15^, they would have to be kept in the hospital for observation. Thus, patients with low risk of hypocalcemia would be prevented from leaving the hospital after the procedure and hospitalization costs would increase. Intact parathyroid hormone (i-PTH) testing done between one and 24 hours after thyroidectomy was recently found to be an effective method in the early detection of patients with symptomatic hypocalcemia requiring supplementation, thus allowing the subjects with low risk of developing this complication to be discharged earlier^16^^,^^17^. Despite the proven efficacy of i-PTH testing in managing total thyroidectomy patients, there is still no consensus as to the when in postoperative care i-PTH testing should be done and whether serial tests or tests associated with serum calcium levels ought to be done.

This study looked into the correlations between i-PTH serum levels measured at different times in post-thyroidectomy care and the occurrence of symptomatic hypocalcemia. The findings of this study and other publications in the literature were used to design an algorithm on the use of i-PTH tests in the clinical setting.

## METHOD

This longitudinal cohort study enrolled 113 consecutive patients submitted to total thyroidectomy and partial to total thyroidectomy conversion procedures in a tertiary care teaching hospital between June of 2006 and December of 2010. The study was registered at CONEP (373972) and approved by the Research Ethics Committee of the institution (CEP 10048).

All patients were tested for i-PTH under anesthetic induction four and 12 hours after surgery with immunometric assay Immulite 2000^®^ (normal threshold between 11 and 67 pg/mL). They were observed clinically for symptom onset (lip and finger extremity paresthesia) and signs of hypocalcemia (Chvostek and Trousseau). Only the subjects with symptomatic hypocalcemia or confirmed low calcium levels (ionic calcium under 1 mmol/L) were given oral calcium and vitamin D supplementation. The patients were informed of the risk of developing hypocalcemia symptoms after discharge and advised to seek medical help before taking the oral supplementation if the symptoms manifested.

Absolute i-PTH values are dichotomic (normal *versus* below normal), and thus the probability of developing hypocalcemia was estimated based on serial testing and tests done four and 12 hours after surgery. In serial testing the lower value obtained between the two tests was considered for risk analysis. The performance of the different PTH testing times as early predictors for hypocalcemia was rated based on their sensitivity, specificity, positive predictive values, and negative predictive values using McNemar's test. Statistical data analysis was performed on software package SAS release 9.2 (SAS Institute, Inc., Cary, N.C, USA).

## RESULTS

Three patients were excluded from the study as they had been taking alendronate and calcium supplements before surgery. Sixteen (14.54%) of the 110 patients included in the study were males and 94 (85.46%) were females; their mean age was 48.25 years. Ninety-six patients (87.2%) underwent total thyroidectomy in one stage, while nine (8.2%) were converted from partial to total thyroidectomy and another five (4.6%) were submitted to neck dissection of the central compartment combined with total thyroidectomy. Pathology testing confirmed the presence of malignant tumors in 55 (50%) patients and benign disease in the remaining subjects. The thirty-one (28.18%) patients with symptomatic hypocalcemia were grouped together and compared to the other 79 (71.82%) patients with normal blood calcium levels. The levels of i-PTH at different points in postoperative care were highly sensitive (90.3% to 96.8%) for symptomatic hypocalcemia, while specificity ranged between 77.2% and 87.3%. No significant differences were found in the sensitivity level of the tests done four or twelve hours after surgery and in serial testing, but the specificity and positive to negative predictive value ratio of i-PTH levels measured twelve hours after surgery were significantly higher than on serial tests (*p* < 0.007). Statistics are summed up in Table 1, Table 2.

**Table 1:** Statistical results.

	Sensitivity	Specificity	PPV	NPV
4-hour PTH	93.6%	78.5%	63%	96.9%
12-hour PTH	90.3%	87.3%	73.7%	95.8%
sPTH	96.8%	77.2%	62.5%	98.4%

PPV (positive predictive value); NPV (negative predictive value).

**Table 2:** McNemar's test comparing 4-hour, 12-hour, and serial i-PTH test results.

	4h PTH vs. 12h PTH	4h PTH vs. sPTH	12h PTH vs. sPTH
Sensitivity	0.936 vs. 0.903	0.936 *vs.* 0.968	0.903 *vs.* 0.968
	(*p* = 1)	(*p* = 1)	(*p* = 0.5)
Specificity	0.785 *vs.* 0.873	0.785 *vs.* 0.772	0.873 *vs.* 0.772
	(*p* = 0.03)	(*p* = 1)	(*p* = 0.007)

The Bonferroni correction states that for multiple comparisons only *p*-values greater than 0.01 are deemed significant.

## DISCUSSION

Parathyroid function may be compromised after thyroidectomy, whether by direct trauma or gland vascular impairment, leading to reduced levels of circulating PTH and consequent drops in blood calcium^18^. Until recently, the detection of patients at high risk for severe hypocalcemia relied on the clinical observation of the signs and symptoms connected to the complication or on drops in blood calcium, usually seen 24-48 hours after surgery^19^^,^^20^. The development of specific immunometric assays to verify the level of i-PTH^21^^,^^22^ aided significantly to the use of parathyroid hormone as an early predictor for hypocalcemia in patients submitted to total thyroidectomy. Due to their short half-life − 3 to 5 minutes - drops in i-PTH can be observed immediately after surgery^23^^,^^24^. Although many studies have looked into the correlations between postoperative i-PTH levels and risk of hypocalcemia, very few tried to establish the obvious resulting clinical applications, once test results and conclusions in these studies vary significantly. Immediate i-PTH levels are generally regarded as the test results from blood collected up to six hours after surgery; late i-PTH is obtained 12 hours after surgery. This study analyzed the differences between immediate, late, and serial i-PTH testing in determining the risk of hypocalcemia. Sensitivity levels were 93.5%, 90.3% and 96.8% in 4-hour, 12-hour, and serial tests respectively.

These findings were consistent with the studies by Soon et al.^25^ (92.3%), Sywak et al.^26^ (90%), and Toniato et al.^27^ (95.85%); these authors found that drops in i-PTH to levels under normal in the assay were well correlated with occurrence of hypocalcemia. These levels of sensitivity are higher than those reported by Scurry et al.^28^ (80%), Di Fabio et al.^29^ (76.2%), and Khafif et al.^30^ (23%); these authors, by their turn, correlated drops in i-PTH greater than 75%-80% from the baseline preoperative values as a risk factor for hypocalcemia.

These findings indicate that using the lower threshold for normal i-PTH levels as a reference to determine the risk of hypocalcemia is more sensitive than calculating percentage drops as proposed by some authors, once the prevalence of severe symptomatic hypocalcemia is higher in patients with subnormal i-PTH levels^31^^,^^32^. No statistically significant differences were found when the sensitivity levels of 4-hour, 12-hour, and serial tests were compared, thus suggesting a strong correlation between the findings obtained from tests done at different times after surgery as shown by Vescan et al.^33^ and Grodski & Serpell^24^.

Observed specificity levels were 78.5% (4-hour PTH), 87.3% (12-hour PTH), and 77.2% (sPTH), showing that tests can be used safely to determine which patients are at a low risk of developing symptomatic hypocalcemia when their postoperative i-PTH levels are normal. The same was seen by Lam & Kerr^34^ in a study enrolling 40 patients, in which no cases of hypocalcemia were observed in subjects with normal postoperative i-PTH levels. The chance of finding subnormal i-PTH levels in patients with hypocalcemia was 4.2 to 7 times greater than the chance of observing altered test results in individuals with normal calcium levels.

This allows patients at a low risk for hypocalcemia to be discharged within 24 hours of surgery, resulting in significant reductions in hospitalization costs as shown by Payne et al.^35^, in a study that revealed a cost reduction of approximately USD 726 per total thyroidectomy patient discharged earlier based on postoperative i-PTH and calcium levels.

**Figure 1:**
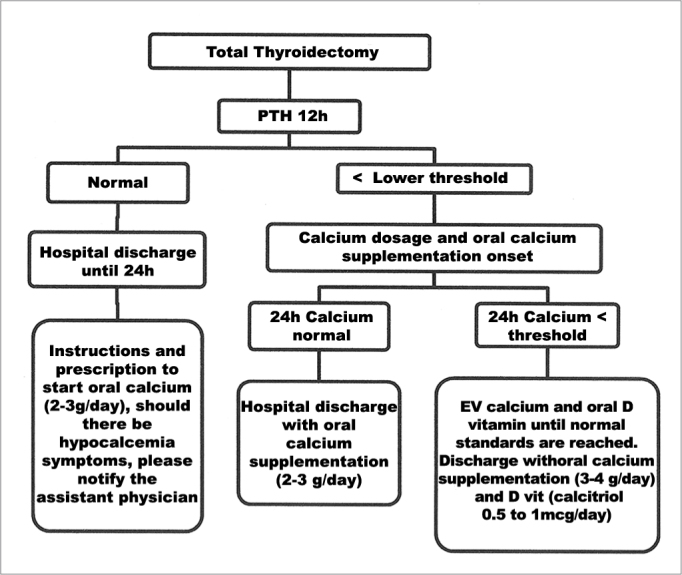
Single test algorithm for 12-hour i-PTH as a predictor for hypocalcemia - PTH - intact parathyroid hormone.

Specificity was greater in the i-PTH tests done 12 hours after surgery (*p* = 0.007). Therefore, we would like to recommend a protocol now in use at our institution, in which one i-PTH test is done to screen patients at a low risk of developing severe symptomatic hypocalcemia after total thyroidectomy as shown in Figure 1.

## CONCLUSION

Intact parathyroid hormone levels are highly sensitive and specific for risk of hypocalcemia. One single test 12 hours after surgery can be used to select patients at a low risk for hypocalcemia and start early treatment for high risk patients.
